# An Aqueous Extract of *Fagonia cretica* Induces DNA Damage, Cell Cycle Arrest and Apoptosis in Breast Cancer Cells via FOXO3a and p53 Expression

**DOI:** 10.1371/journal.pone.0040152

**Published:** 2012-06-27

**Authors:** Matt Lam, Amtul R. Carmichael, Helen R. Griffiths

**Affiliations:** 1 School of Life and Health Sciences, Aston University, Birmingham, United Kingdom; 2 Russell Hall, Dudley Group Hospitals, Dudley, United Kingdom; University of Medicine and Dentistry of New Jersey, United States of America

## Abstract

**Background:**

Plants have proved to be an important source of anti-cancer drugs. Here we have investigated the cytotoxic action of an aqueous extract of *Fagonia cretica*, used widely as a herbal tea-based treatment for breast cancer.

**Methodology/Principal Findings:**

Using flow cytometric analysis of cells labeled with cyclin A, annexin V and propidium iodide, we describe a time and dose-dependent arrest of the cell cycle in G0/G1 phase of the cell cycle and apoptosis following extract treatment in MCF-7 (WT-p53) and MDA-MB-231 (mutant-p53) human breast cancer cell lines with a markedly reduced effect on primary human mammary epithelial cells. Analysis of p53 protein expression and of its downstream transcription targets, p21 and BAX, revealed a p53 associated growth arrest within 5 hours of extract treatment and apoptosis within 24 hours. DNA double strand breaks measured as γ-H2AX were detected early in both MCF-7 and MDA-MB-231 cells. However, loss of cell viability was only partly due to a p53-driven response; as MDA-MB-231 and p53-knockdown MCF-7 cells both underwent cell cycle arrest and death following extract treatment. p53-independent growth arrest and cytotoxicity following DNA damage has been previously ascribed to FOXO3a expression. Here, in MCF-7 and MDA-MB-231 cells, FOXO3a expression was increased significantly within 3 hours of extract treatment and FOXO3 siRNA reduced the extract-induced loss of cell viability in both cell lines.

**Conclusions/Significance:**

Our results demonstrate for the first time that an aqueous extract of *Fagonia cretica* can induce cell cycle arrest and apoptosis via p53-dependent and independent mechanisms, with activation of the DNA damage response. We also show that FOXO3a is required for activity in the absence of p53. Our findings indicate that *Fagonia cretica* aqueous extract contains potential anti-cancer agents acting either singly or in combination against breast cancer cell proliferation via DNA damage-induced FOXO3a and p53 expression.

## Introduction

Following genotoxic stress, an intact DNA damage response (DDR) is necessary to eliminate lethal and tumorigenic mutations. The DDR is a network of molecular signalling events that control and coordinate DNA repair, cell cycle arrest and apoptosis [1]. An impairment in the DNA damage response represents a double-edged sword, where on one side loss of repair mechanisms can drive tumorigenesis and on the other, can affect sensitivity to genotoxic chemotherapy [2], [3].

The tumour suppressor protein, p53, plays a pivotal role in regulating the cellular response to stress and damage signals. Several of the cell signalling pathways involved in the DDR and cell differentiation converge with p53 [4] and loss of p53 functionality is common in more than 50% of cancers [5]. In response to stress signals, post-translational modifications of p53 such as phosphorylation, drive its nuclear translocation and subsequent target gene transcription [6], [7]. Normally, upon DNA damage, p53 is rapidly stabilised by the DNA damage sensor, ATM, via phosphorylation of serine-15 within the p53 N-terminus activation domain [8]. Consequently, dissociation of the MDM2-p53 repressor complex, prevents monoubiquitination of p53 and its degradation [9], [10]. This in turn increases p53 half-life and activates its transcriptional program [11].

Important p53 transcriptional targets include cell cycle control genes such as p21 (WAF1/CIP1), 14-3-3σ and cyclin G, and pro-apoptotic genes such as BAX [12]. The cyclin dependent kinase inhibitor, p21, is a direct regulator of the cell cycle, inducing growth arrest in G1-phase of the cell cycle by binding to and inhibiting the activity of cyclinD-CDK2/4 complexes [13]. Increased transcription and translation of p21 prevents cyclinD-CDK2/4 mediated phosphorylation of retinoblastoma protein (pRb), thus, inhibiting E2F transcriptional activity and cell cycle progression to S-phase [14].

However, p53-independent growth arrest and cell death has also been observed following ionizing radiation and DNA damage (the cell death machinery governed by p53 [15]. Recently, it has been shown that in response to DNA damage, the transcription factor FOXO3a is vital to initiating growth arrest [16]. Moreover, induction of DNA damage by ionizing radiation, activates FOXO3a and increases its nuclear translocation. The FOXO3a-dependent activation of Bim and Fas ligand expression is associated with induction of apoptosis, and is observed independently of p53, highlighting a potential FOXO3a-mediated response to DNA damage [17]. As well as this, FOXO3a is a regulator of metabolic homeostasis, via its interaction with Akt and AMPk signaling pathways [18]. Pharmacological modulation of these pathways has been shown to induce cell death in cancer cells via FOXO3a-dependent mechanisms [19], [20].

Targeting the cell cycle to induce arrest pharmacologically is known to be effective in restricting tumour growth in vitro and in vivo [21], [22], particularly in transformed cells that have an aberrant response to genotoxic and cellular damage [23]. We have investigated the potential for *Fagonia cretica* to inhibit the growth of breast cancer cells via a DNA damage driven response. *Fagonia cretica* is a herbaceous plant found in arid, desert regions of Pakistan, India, Africa and parts of Europe. It is a common plant used in local medicine as a herbal tea to remedy breast cancer. However, mechanism(s) of action for *Fagonia cretica* extracts on breast cancer cells have not been investigated. Herein, we show that an aqueous extract of *Fagonia cretica* induces growth arrest and apoptosis in human breast cancer cells by inducing DNA damage and activation of p53 and FOXO3a.

## Results

### Extract treatment induces cell cycle arrest and apoptosis in MCF-7 cells

**Figure 1:**
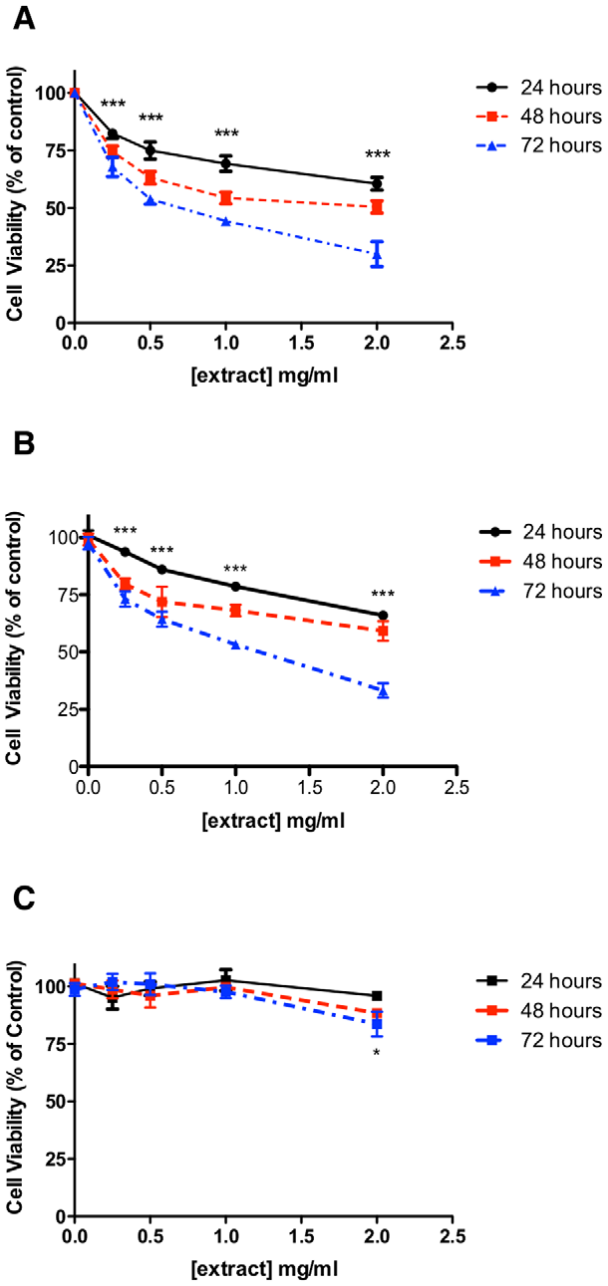
Fagonia cretica extract treatment reduces breast cancer cell viability. (A) MCF-7, (B) MDA-MB-231 and (C) HMEpC cells were treated with up to 2mg/ml aqueous extract for up to 72 hours prior to analysis of cell viability by MTT assay. Data denoted * (p<0.05) and *** (p<0.001) are significant at all time points compared to untreated control analysed by one-way ANOVA with Dunnett's multiple comparison post test. Data denoted # (p<0.05) is significant at 72 hours only compared to untreated control analysed by one-way ANOVA with Dunnett's multiple comparison post test. All data is representative of at least three independent experiments performed in triplicate.

In order to determine whether an aqueous extract of *Fagonia cretica* had any cytotoxicity towards normal and breast cancer cells in vitro, we tested its effects on MCF-7 and MDA-MB-231 cell viability and cell cycle status, alongside HMEpC. Extract treatment in the concentration range 0-2mg/ml over 72 hours induced a significant time and dose dependent reduction in MCF-7 cell viability (Figure 1A) with an approximate 75% reduction in cell viability after 72 hours treatment with 2mg/ml aqueous extract. Similar treatment of MDA-MB-231 cells also induced a significant time and dose dependent decrease in cell viability (Figure 1B), with an approximate 67% reduction in cell viability after 72 hours with 2mg/ml extract. The cytotoxic/static effect of extract treatment was more pronounced in MCF-7 cells [IC_25_ = 0.43mg/ml] than MDA-MB-231 cells [IC_25_ = 1.01mg/ml] at 24h, although the concentration of active(s) within the extract is unknown. In parallel, it was shown that an IC_25_ could not be reached and only extract treatment of 2mg/ml had any significant effect on HMEpC viability after 72 hours, with an approximate 20% reduction in cell viability (Figure 1C). This suggests greater activity of extract towards human breast cancer cell lines.

**Figure 2:**
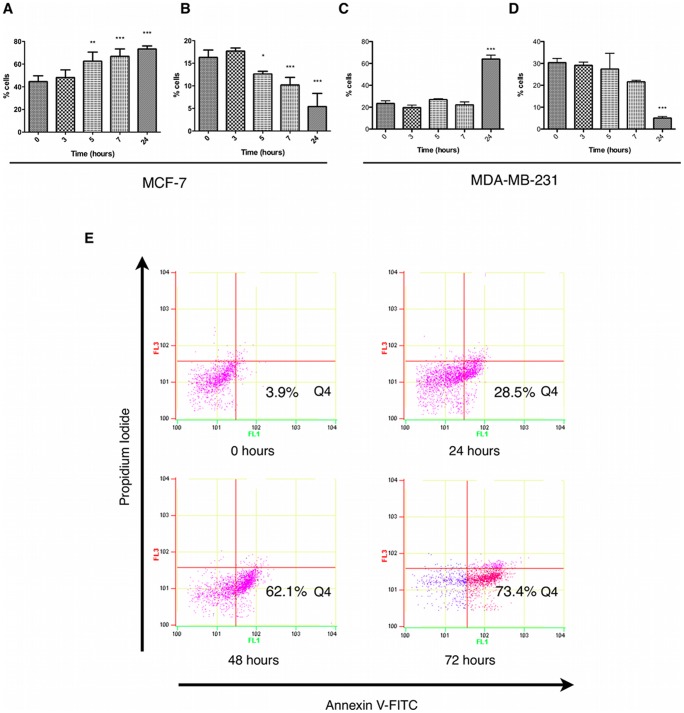
Fagonia cretica extract induced cell cycle arrest and apoptosis in human breast cancer cells. MCF-7 and MDA-MB-231 cells were treated with 2mg/ml extract for up to 24 hours prior to cell cycle analysis using cyclin A/propidium iodide staining. (A) G0/G1 MCF-7, (B) G2 MCF-7, (C) G0/G1 MDA-MB-231, (D) G2 MDA-MB-231. (E) MCF-7 cells were treated with 2mg/ml extract for up to 72 hours prior to detection of apoptosis as annexin V positive/propidium iodide negative stained cells (Q4). Data denoted * (p<0.05), ** (p<0.01) and *** (p<0.001) are significant compared to controls (time  = 0) analysed by one-way ANOVA with Dunnett's multiple comparison post test (n = 3 independent experiments). Blots are representative of at least three independent experiments.

As hyper-proliferation is a characteristic common to tumour cells, which as a result are more susceptible to cell cycle modulation, we assessed the effects of extract treatment on MCF-7 and MDA-MB-231 cell cycle using flow cytometry. We found that extract treatment could induce a significant increase in cells expressing low levels of cyclin A associated with G1-phase of the cell cycle (Figure 2A) with a parallel reduction in G2-phase cells expressing higher levels of cyclin A (Figure 2B and data not shown) after 5 hours treatment in MCF-7 cells. This suggests a potential blockade of cell cycle progression at the G1/S checkpoint. Extract-treated MDA-MB-231 cells also exhibited a G1 arrest (Figure 2C) and a parallel reduction in G2-phase cells (Figure 2D) but induction of G1 arrest was delayed until after 24 hours treatment. Cell cycle checkpoints represent an intersection of cell survival and cell death where conditions for successful interphase and mitosis have to be favourable for complete cell division or the cell commits to death. In accordance with this, analysis of apoptosis by flow cytometry, was used to determine the effects of extract treatment on apoptotic induction in MCF-7 cells. The results revealed a significant increase of annexin V binding in PI negative cells, representative of apoptosis, after 24 hours treatment which increased through to 72 hours (Figure 2E).

### Cell cycle arrest is associated with activation of the DNA damage response

**Figure 3:**
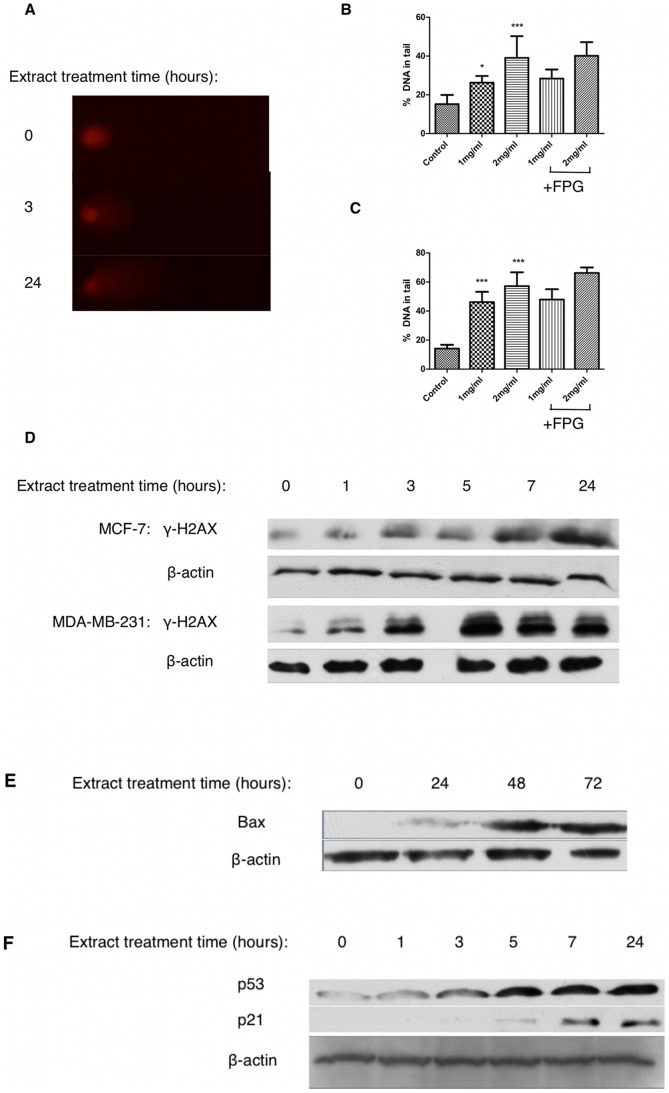
Fagonia cretica extract treatment induces double strand breaks in human breast cancer cells. MCF-7 cells were treated with up to 2mg/ml extract for (B) 3 or (C) 24 hours prior to detection of DNA damage using the comet assay with and without FPG protein incubation. (A) Representative comets after 0, 3 and 24 hour exposure to 2mg/ml extract. (D) MCF-7 and MDA-MB-231 cells were treated with 2mg/ml extract for 24 hours prior to SDS-PAGE and western blot detection of γ-H2AX and β-actin. MCF-7 cells were treated with 2mg/ml extract for up to 24 hours prior to SDS-PAGE and western blot detection of (E) BAX (F) p53, p21 and β-actin. Data denoted * (p<0.05) and *** (p<0.001) are significant compared to control analysed by one-way ANOVA with Dunnett's multiple comparison post test.

**Figure 4:**
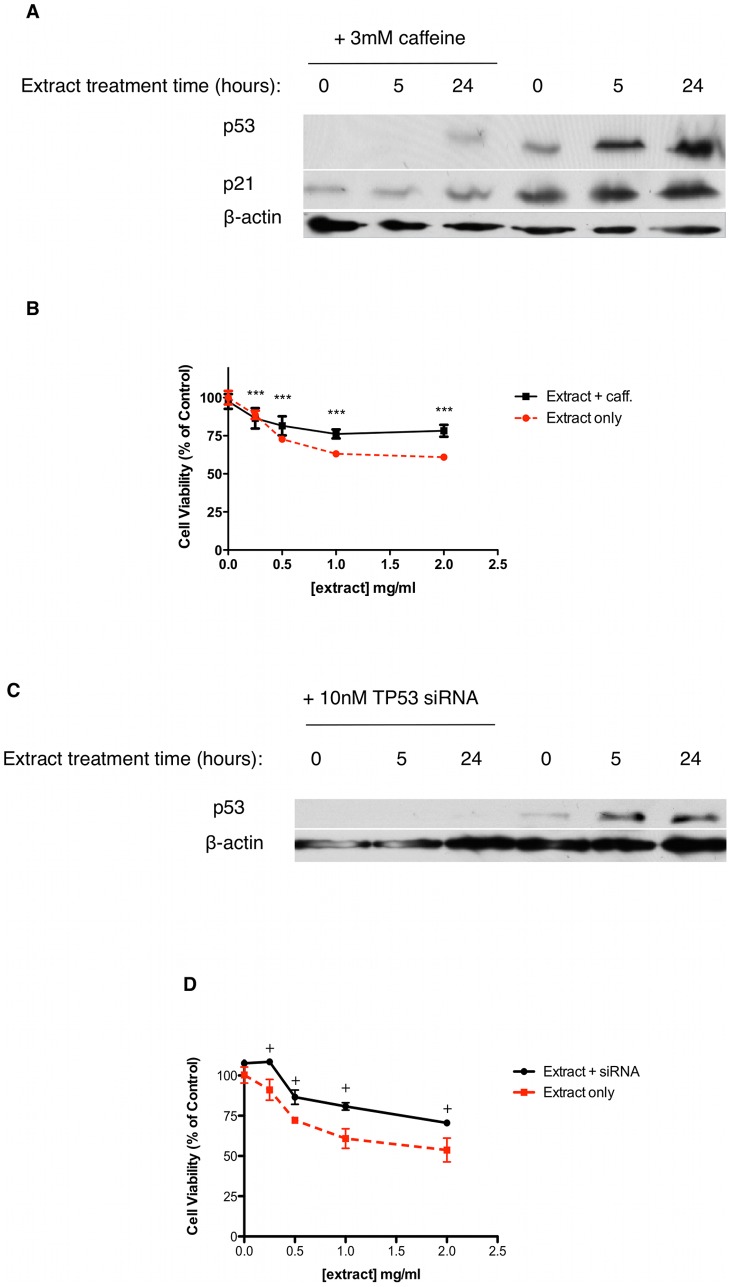
Fagonia cretica extract-induced p53 expression occurs as a result of activation of the DNA damage response and is only partly responsible for loss of cell viability. (A, B) MCF-7 cells were treated with and without 3mM caffeine (caff) for 60 minutes prior to up to 2mg/ml extract treatment for up to 24 hours. Expression of p53 and β-actin was determined by SDS-PAGE and western blot. Cell viability was determined by MTT assay. (C, D) MCF-7 cells were transfected with 10nM TP53 siRNA for 24 hours prior to up to 2mg/ml extract treatment for up to 24 hours. Expression of p53 and β-actin was determined by SDS-PAGE and western blot. Cell viability was determined by MTT assay. Data denoted *** (p<0.001) is significant compared to control analysed by one-way ANOVA with Dunnett's multiple comparison post test. Data denoted ♦ (p<0.001) is significant compared to ‘no-siRNA’ as analysed by two-way ANOVA with Bonferroni's multiple comparison post test. All data is representative of at least three independent experiments.

Cell cycle arrest is initiated via activation of the DNA damage response following genotoxic stress. We used the comet assay to detect the presence and level of DNA strand breaks in extract-treated MCF-7 cells. Our results indicate that extract treatment induces a dose dependent increase in DNA damage, measured as % DNA present in a comet tail after 3 hours (Figures 3A and 3B), that is sustained through at least 24 hours (Figures 3A and 3C). Post-treatment incubation with FPG, a protein that excises 8-oxo-dG, did not alter the level of DNA damage seen suggesting that DNA damage is non-oxidative (Figure 3B and 3C). Furthermore cell survival in the presence of extract was not affected by pretreatment with the antioxidant N-acetyl-cysteine (data not shown). Treatment of MCF-7 and MDA-MB-231 cells for up to 24 hours with 2mg/ml extract induced double strand breaks to DNA as shown by increased levels of γ-H2AX over time (Figure 3D). Induction of the DDR involves sensors such as ATM relaying a signal to transducers such as p53 to exert cell cycle arrest via their transcriptional targets. Immunoblotting of MCF-7 cell lysates after treatment with 2mg/ml extract for up to 24 hours revealed a significant increase in p53 protein expression as well as increased expression of its transcriptional targets, p21 (Figure 3E) and BAX (Figure 3F), suggesting that extract treatment is modulating p53-directed cell cycle arrest and apoptosis. In order to determine if activation of p53 is linked to the presence of DNA damage we used caffeine, a known inhibitor of ATM/ATR [24], in combination with extract and assessed p53 and p21 protein expression. Our results show that inhibition of the DNA damage sensors ATM/ATR with caffeine prevents the increased expression of p53 and p21 caused by extract treatment (Figure 4A). Furthermore, caffeine attenuated some but not all of the extract-induced cytotoxicity (Figure 4B). Taken together, these results suggest that extract treatment induces double strand breaks, which stabilises p53 in an ATM/ATR dependent manner, thus increasing p53 dependent transcription of p21 and inducing cell cycle arrest.

### Activation of p53 is not essential for loss of cell viability

We have shown that extract treatment of MCF-7 cells induces DNA damage leading to activation of p53, cell cycle arrest and apoptosis. The tumour suppressor p53 is mutant in over 50% of cancers and its loss of function has been shown to be a key event in neoplasia. We have already shown that the mutant-p53 breast cancer cell line MDA-MB-231 is susceptible to extract treatment and that inhibition of extract-induced p53 expression in MCF-7 cells associates with improved cell survival in response to extract but does not abrogate extract effect completely. In order to verify the role of p53, we successfully transfected MCF-7 cells (wild-type p53) with *TP53* siRNA and treated them with extract for 24 hours. Our results show that siRNA knockdown could significantly reduce an extract-induced increase in p53 expression while reducing loss of cell viability (Figures 4C and 4D). However, this did not fully alleviate the effect of extract treatment, providing further evidence that factors other than p53 are contributing to the loss of cell viability seen in MCF-7 cells. Taken together, this data suggests that while p53 activation is occurring in response to DNA damage, the overall effect of cell cycle arrest and cell death appear to remain intact, albeit reduced. This suggests that activation of p53 is important but not essential for cytotoxic activity of extract treatment.

### Extract-induced cytotoxicity is dependent on FOXO3a expression

**Figure 5:**
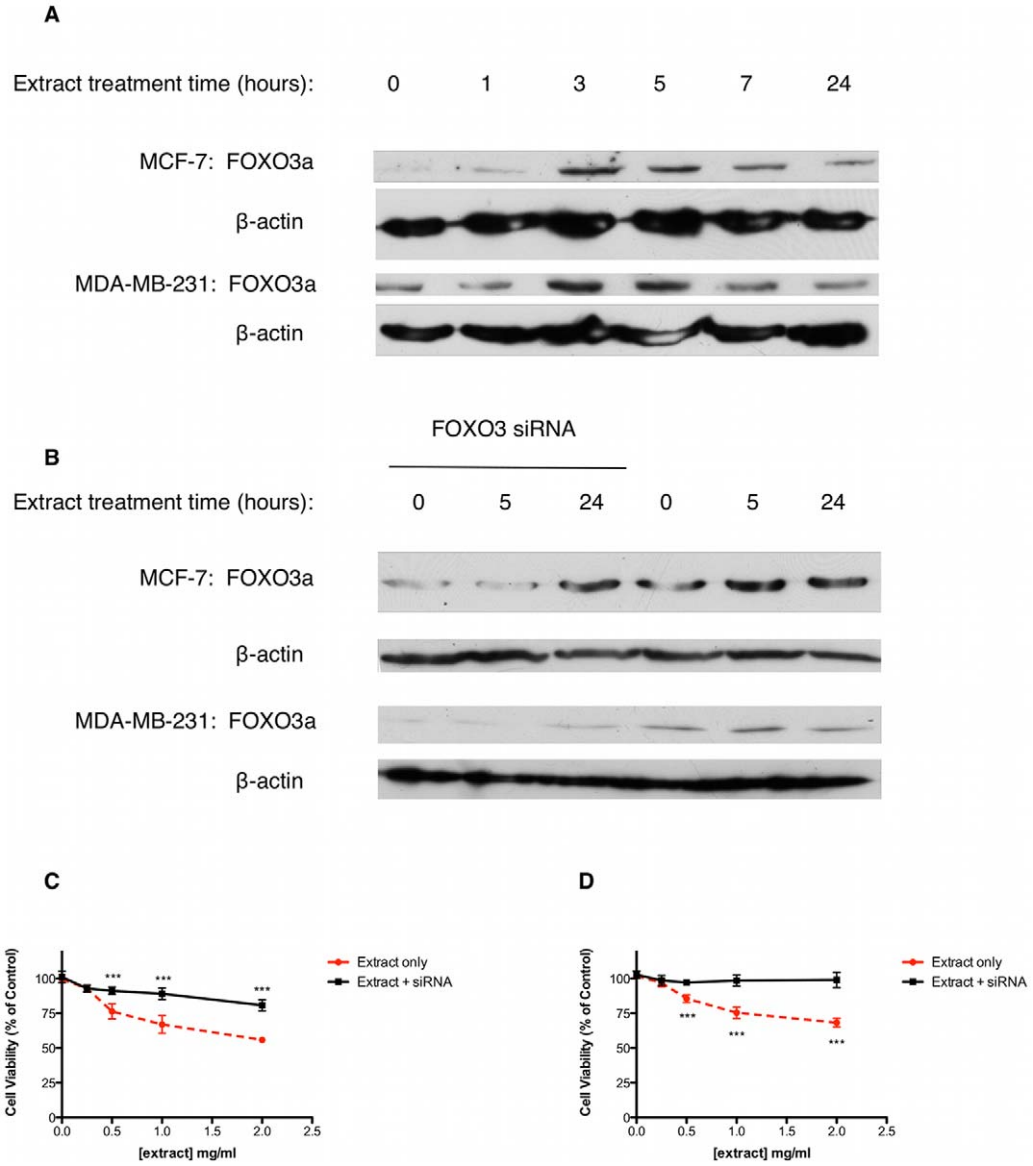
Fagonia cretica extract-induced cytotoxicity is dependent on FOXO3a expression. (A) MCF-7 and MDA-MB-231 cells were treated with 2mg/ml extract for up to 24 hours prior to FOXO3a protein expression analysis by SDS-PAGE and western blot. β-actin was used as a loading control. (C) MCF-7 and (D) MDA-MB-231 cells were treated with up to 2mg/ml extract for 24 hours with and without FOXO3 siRNA transfection (B). Cell viability was determined by MTT assay. Data denoted * (p<0.05), ** (p<0.01) and *** (p<0.001) are significant compared to untreated control as analysed by one-way ANOVA with Dunnett's multiple comparison post test. Data is representative of three independent experiments.

The FOX class ‘O’ (FOXO) transcription factors are involved in the cellular stress response and regulate cell cycle progression and apoptosis. The FOXO member FOXO3a has been shown to be vital in the initiation of cell cycle arrest, as well as being involved in DNA damage mediated apoptosis, independently of p53. It is also known that FOXO3a is an important tumour suppressor and is under-expressed in many breast cancers. Therefore, we hypothesised that extract treatment may increase FOXO3a expression in MCF-7 and MDA-MB-231 cells resulting in p53-independent cytotoxicity. Our results show that FOXO3a expression in both MCF-7 and MDA-MB-231 cells is increased after 3 hours treatment with 2mg/ml extract (Figure 5A). In both cell lines this increase peaks at 5 hours and tapers off towards 24 hours treatment. To determine whether or not an extract-induced increase in FOXO3a was required for cytotoxicity, MCF-7 and MDA-MB-231 cells were successfully transfected with FOXO3 siRNA, prior to extract treatment. Knockdown of FOXO3a expression (Figure 5B and Figure S2) in MCF-7 cells significantly reduced extract-induced loss of cell viability compared to extract treatment alone at concentrations above 0.5mg/ml (Figure 5C). Extract-induced loss of cell viability was still significant after FOXO3a siRNA transfection probably due to the p53-mediated effects described previously. In comparison, knockdown of FOXO3a expression (Figure 5B and Figure S2) in MDA-MB-231 cells completely abrogated loss of cell viability, in response to extract treatment (Figure 5D).

## Discussion

In this study we report mechanisms of *Fagonia cretica* aqueous extract-induced cytotoxicity in breast cancer cells. Local medical practitioners use *Fagonia cretica* for treating a wide variety of ailments, including cancer [25]. This substance is well tolerated and does not exhibit adverse effects like vomiting, diarrhea or alopecia, which are common side effects of standard cytotoxic therapy. To the authors' best knowledge, this study is the first time that cytotoxic activity towards human breast cancer cell lines has been described. Herein, we have shown that an aqueous extract of *Fagonia cretica* is able to induce cell cycle arrest and apoptosis in wild type p53 MCF-7 and mutant p53 MDA-MB-231 cells, while only exerting a limited effect on primary HMEpC at high concentrations and extended treatment time. We have also demonstrated that cell cycle arrest may be associated with induction of DNA damage and in MCF-7 cells, via activation of the ATM/p53-mediated DNA damage response. Interestingly, the requirement of p53 activation is not essential for cytotoxicity, as we have shown with siRNA p53 knockdown in extract-treated MCF-7 cells, and the significant treatment effects on mutant-p53 MDA-MB-231 cells. In contrast, extract-induced cytotoxicity is shown to be dependent on induction of FOXO3a expression, in both cell types.

Induction of cell cycle arrest occurs in response to various stresses including DNA damage [26]. Stabilisation and activation of p53 can occur as a result of serine-15 phosphorylation by ATM/ATR in the presence of DNA damage [27]. This in turn allows for p53 nuclear translocation and activation of transcriptional targets such as p21 and BAX to regulate cell cycle control and apoptosis [28]. According to our results, extract treatment of MCF-7 cells induced arrest in G1-phase of the cell cycle and triggered apoptosis, which may be controlled by p53-mediated transcription of the CDK-inhibitor p21 and pro-apoptotic BAX. This result is consistent with the literature on tamoxifen which describes G1-arrest induced by DNA damage in cancer cells [29]. Blockade of extract-induced p53 expression using a phamacological inhibitor of ATM/ATR, caffeine, attenuated loss of cell viability in MCF-7 cells. This suggests activation of the DNA damage response is driving p53-mediated effects in extract-treated MCF-7 cells. Indeed, it was further shown that extract treatment may induce double strand breaks in MCF-7 cells, detectable by comet assay and by the presence of γ-H2AX, however, other forms of DNA damage can increase comet assay results and γ-H2AX expression. This DNA damage response pathway is well characterised and provides a potential mechanism by which extract treatment induces cell cycle arrest and apoptosis in MCF-7 cells [30], [31]. Mutations in p53 that generate a non-functional phenotype are common in tumours [5], and although frequency is lower in breast tumours than in other tumour types, mutant status is associated with a more aggressive disease and mediates tumour cell survival [32], [33]. It is therefore important that drugs are developed that can specifically target cancer cells independent of their p53 status. We used siRNA against *TP53* to knockdown p53 expression in p53 wild-type MCF-7 cells and then treated the cells with aqueous extract. Inhibition of p53 expression did reduce the cytotoxic effect of treatment but did not fully abrogate the loss of cell viability due to extract treatment. This suggests that p53 mediated cytotoxicity is an additional effect seen in cells that carry a functional form of p53 but is not vital to the treatment effect. We confirmed this effect in MDA-MB-231 breast cancer cells, which carry a mutant, non-functional form of p53. Indeed, we demonstrated that extract-induced cytotoxicity in MDA-MB-231 cells is less than in MCF-7 cells but remains significant at 24h. It has been shown previously that cells can arrest in the G1-phase of the cell cycle independent of the p53-p21 axis [34], and also that apoptosis can be initiated without p53 activation [35]. Extract-treated MDA-MB-231 cells also underwent G0/G1 arrest but induction was delayed until 24 hours providing further support for the notion that p53 expression in MCF-7 cells drives extract-induced growth arrest. It has been shown previously that p53 functionality governs kinetics of cell cycle arrest in response to DNA damage thus providing a mechanism by which absence of p53 could delay onset of cell cycle arrest [36]. It was evident that double strand breaks were induced in both MCF-7 and MDA-MB-231 cells upon extract treatment suggesting a shared mechanism driving cell death. Indeed, it has been shown recently that in response to DNA damage, p53-mutant cells undergo p53-independent cell cycle arrest and apoptosis, offering a significant therapeutic strategy for p53-mutant cancers [37].

Members of the forkhead class ‘O’ (FOXO) family of transcription factors have been implicated in tumorigenesis [38]. In particular FOXO3a has been shown to function as a tumour suppressor in ERα-positive and negative breast cancers [39], [40]. It has also been reported recently that nuclear localisation of FOXO3a and subsequent transcriptional activity is a marker of good prognosis among breast cancer patients [41]. As well as this, FOXO3a has been show to regulate cell cycle arrest and apoptosis in response to DNA damage, via activation of transcriptional targets such as Bim, p27 and Fas-L [17], [42]. We report here that FOXO3a expression is increased in both MCF-7 and MDA-MB-231 cells in response to extract treatment. Furthermore, suppression of extract-induced FOXO3a expression using FOXO3 siRNA, attenuated cytotoxicity in MCF-7 cells and completely abrogated cytotoxicity in MDA-MB-231 cells. Interestingly, levels of FOXO3a protein expression correlate with time points where significant DNA damage is exhibited, suggesting FOXO3a expression may be directly linked to DNA damage. This provides evidence for FOXO3a-dependent cell cycle arrest and death in breast cancer cells that works independently of p53 following extract treatment. Although FOXO3a involvement in oxidative stress and survival signal withdrawal-induced transcriptional activity is well documented [43], the role of FOXO3a in response to DNA damage, is relatively unclear. FOXO3a is activated as a survival response to energy depletion and can drive autophagy and apoptosis [44]. Indeed, treatment with *Fagonia cretica* reduced ATP levels significantly in MDA-MB-231cells within 3 hours (data not shown). Energy depletion can occur as a result of excessive PARP activation due to DNA damage [45]. Therefore, it is possible that DNA damage may induce a metabolic stress, which directly activates FOXO3a. Furthermore, FOXO3a driven transcription of DNA repair genes, including PARP, may further deplete cellular NAD+ and ATP and lead to cell death [42], [46].

Why do HMEpC remain viable following extract treatment compared to MCF-7 or MDA-MB-231 cells? Cytotoxic agents are known to induce DNA damage in normal cells as well as cancer cells. However, fast growing cells are more susceptible to DNA damaging agents due to the greater probability of more sites being exposed on DNA within replicative cycles and, in addition, cancer cells frequently have defective repair pathways resulting in DNA damage being sustained. While normal cells may also up-regulate FOXO3a in response to energy depletion and DNA damage, they are less dependent on glycolytic metabolism than cancer cells. They may be less energetically challenged in the presence of *Fagonia cretica* because of the potential to use oxidative phosphorylation as an additional energy source.

### Conclusion

We have shown here for the first time that an extract of *Fagonia cretica* induces cell cycle arrest and apoptosis in two phenotypically distinct breast cancer cell lines. Extract activity involves DNA damage and p53-induction but is not fully dependent on p53 functionality. In addition, extract treatment induces FOXO3a expression which may be attributed to DNA damage directly or induction of DNA repair pathways. We also demonstrated that FOXO3a expression is required for extract activity in the absence of functional p53. This provides a novel mechanism by which an aqueous extract of *Fagonia cretica*, used extensively in Pakistan, can kill breast cancer cells *in vitro*. However, the molecular composition of the bioactive(s), remains to be determined.

## Materials and Methods

### Cell culture

MCF-7 (HPA Cultures, UK) and MDA-MB-231 human breast cancer epithelial cells (HPA Cultures, UK) were cultured in RPMI 1640 with stable glutamine (PAA, UK) supplemented with 10% FCS and 1% penicillin/streptomycin (50U/ml) and incubated at 37°C with 5% C0_2_. HMEpC cells (Invitrogen, UK) were cultured in mammary epithelial growth medium (Invitrogen, UK) supplemented with growth supplements (Invitrogen, UK; bovine pituitary extract 0.4% v/v, bovine insulin 5μg/ml, hydrocortisone 0.5μg/ml, human epidermal growth factor 3ng/ml) and 1% penicillin/streptomycin (50U/ml) and incubated at 37°C with 5% CO_2_. Cells were seeded at a density of 2×10^5^ cells per ml in T75 and allowed to reach 80–90% confluence over 7 days prior to sub-culture.

### Fagonia cretica extract preparation and cell treatment

An aqueous extract was prepared by soaking dried plant material (20g) in 500ml d.H_2_O at 70°C for 5 hours with constant agitation. The extract was filtered with Fisherbrand filter paper (Fisher Scientific, FB59020, UK) to remove solids before being subjected to liquid-liquid partition with 3 times equal volumes of hexane. The aqueous phase was dried under vacuum and stored at 4°C.

Cells were treated for up to 24 hours with 2mg/ml extract prior to MTT assay or cell lysate collection for SDS-PAGE and western blot. For caffeine pre-treatment experiments, cells were incubated with 3mM caffeine for 60 minutes, prior to extract treatment.

### siRNA interference

Validated Silencer TP53 siRNA (Ambion, UK) was used to knockdown p53 expression in MCF-7 cells. Sequences were: sense 5′-GGGUUAGUUUACAAUCAGC(dtdt)-3′ and antisense 5′-GCUGAUUGUAAACUAACCC(dtdt)-3′. Efficiency of siRNA knockdown was monitored for effects on cell viability (MTT) and p53 expression (immunoblot). Transfection controls used Silencer® Negative Control (Ambion. UK, 4404021). 10nM of siRNA oligonucleotides was incubated in Opti-MEM (Invitrogen, UK) at a ratio of 1∶50 with 1% v/v lipofectamine RNAiMAX (Invitrogen, UK) and incubated at room temperature for 20 minutes. Cells were seeded at a density of 1×10^5^ cells per ml in antibiotic-free RPMI to tissue culture plates containing siRNA-lipofectamine duplexes and incubated in cell culture conditions for 24 hours.

Validated Silencer FOXO3 siRNA (Ambion, UK) was used to knockdown expression MCF-7 and MDA-MB-231 cells. Sequences were: sense 5′-GGCUCCUCCUUGUACUCAAtt-3′ and antisense 5′-UUGAGUACAAGGAGGAGCCtg-3′ Efficiency of siRNA knockdown was monitored for effects on cell viability (MTT) and p53 expression (immunoblot). Transfection controls used Silencer® Negative Control (Ambion. UK, 4404021). Methods of siRNA knockdown was as per TP53 siRNA transfection.

### Cell viability – MTT assay

Cell viability was determined using the MTT (3-(4,5-dimethylthiazol-2-yl)-2,5-diphenyltetrazolium bromide) colorimetric assay. Cells were seeded at a density of 2×10^5^ cells per ml in tissue culture plates and allowed to adhere overnight. Cells were treated with extract (0–2mg/ml) for up to 72 hours prior to addition of MTT reagent (0.5mg/ml in PBS) and incubation for 4 hours at 37°C with 5% CO_2_. Cells were lysed and formazan solublised over 24 hours with lysis buffer (0.7M SDS, 50% DMF, pH 4.7) and results determined using a spectrophotometer at 570nm.

### Cell cycle analysis – Flow cytometry

Cell cycle status was determined using flow cytometric analysis and gating against cellular cyclin A levels in combination with cellular DNA content (Figure S1). Cells were seeded at a density of 2×10^5^ cells per ml in tissue culture plates and allowed to adhere overnight. Cells were treated with extract (2mg/ml) for up to 24 hours before cells were washed twice with 1ml PBS and scraped into 1ml PBS. Cells were pelleted (300*g*, 5 minutes) and fixed (1% formaldehyde in PBS) for 20 minutes at room temperature. Cells were pelleted (300*g*, 5 minutes) and washed once with 1ml PBS before being re-suspended in permeabilisation buffer (0.25% Triton X-100, 0.5% BSA, 50mM PBS) and stored for 10 minutes at room temperature. Cells were stained with either anti-cyclin A-FITC (BD Pharmingen, UK) or FITC mouse IgE isotype control for 50 minutes at room temperature in the dark followed by propidium iodide staining (10μg/ml, 1% sodium citrate) for 10 minutes. Cells were washed in permeabilisation buffer before immediate analysis by flow cytometry (Beckman Coulter) using FL1 (Em: 525nm) and FL3 (Em: 670nm).

### Detection of apoptosis – Flow cytometry

Induction of apoptosis was assessed using flow cytometric analysis of outer membrane phosphatidylserine translocation. Cells were seeded at a density of 2×10^5^ cells per ml in tissue culture plates and allowed to adhere overnight. Cells were treated with extract (2mg/ml) for up to 72 hours before washing twice with 1ml PBS and scraping into 1ml PBS. 1×10^5^ cells per sample were stained with annexin V-FITC (Abcam, UK) and propidium iodide (0.005%) for 5 minutes. Cells were analysed immediately by flow cytometry using FL1 (Em: 525nm) and FL3 (Em: 670nm).

### DNA damage detection – Comet assay

Presence of DNA strand breaks was measured using the single-cell gel electrophoresis comet assay. Cells were seeded at a density of 2×10^5^ cells per ml in tissue culture plates and allowed to adhere overnight. Cells were treated with 2mg/ml extract for up to 24 hours before washing twice with 1ml PBS and scraping into 1ml PBS. 2×10^4^ cells were embedded in type VII-A low melting point agarose on a microscope slide before lysis (100mM Na_2_.EDTA, 2.5M NaCl, 10mM Tris-HCl, 1% Triton-X) for 1 hour at 4°C in the dark. Cells were incubated with and without FPG protein from *E. coli* (20U/ml) for 30 minutes at 37°C. Slides were submerged in ice cold electrophoresis buffer (0.3M NaOH, 1mM Na_2_.EDTA) for 40 minutes at 4°C in the dark before being subjected to electrophoresis (25V, 300mA) for 30 minutes at 4°C in the dark. Slides were neutralised (0.4M Tris-HCl, pH 7.5) and washed with d.H_2_O before staining with propidium iodide (150μM PI, 10mM KH_2_PO_4,_ 150mM NaCl) for 20 minutes at room temperature in the dark. Comets were visualised and analysed using COMETscore (http://www.autocomet.com). At least 50 comets were scored per slide and strand breaks reported as percentage DNA in comet tail.

### Protein characterisation – Western blot

Specific protein expression was measured by western blot. Cells treated with 2mg/ml extract for up to 72 hours were collected in lysis buffer (150mM NaCl, 1% Triton X-100, 0.5% SDS, 50mM Tris-base, 0.1% protease inhibitor cocktail (AEBSF 104mM, Aprotinin 80μM, Bestatin 4mM, E-64 1.2mM, Leupeptin 2mM, Pepstatin A, 1.5mM) pH 8.0) and DNA was sheared by passing 10 times through a 25-gauge needle. Protein concentration was determined using BCA reagent and 25μg total protein used for western blot. Samples were prepared with Laemmli buffer (1∶1 v/v) and heated at 95°C for 5 minutes before loading into SDS-PAGE gels (16%). SDS-PAGE was achieved in running buffer (25mM Tris-base, 190mM glycine, 0.1% SDS) for 120 minutes at 115V. Separated proteins were transferred to Hybond™-P membrane (Amersham, UK) in transfer buffer (25mM Tris-Base, 190mM glycine, 20% methanol) for 70 minutes at 240mA. Membranes were blocked with 3% BSA in TBS-Tween (50mM Tris-base, 200mM NaCl, 0.05% Tween-20, pH 7.5) overnight at 4°C. Membranes were probed with primary antibody for 2 hours in TBS-Tween (0.2% BSA) followed by probing with secondary detection antibody. Primary antibodies were anti-p53 (Abcam, ab2433), anti-p21 (Abcam, ab7960), anti-BAX (Abcam, ab7977), anti-γH2AX (Sigma, H5912), anti-FOXO3a (Abcam, ab47285) and loading control anti-β-actin (Sigma, A5441). Secondary antibodies were HRP conjugated anti-mouse IgG (Sigma) and anti-rabbit IgG (Abcam). Visualisation of bound secondary antibody was by enhanced chemiluminescence.

### Reagents

All reagents are from Sigma unless otherwise stated.

### Statistics

Statistical analyses were carried out using one-way ANOVA or two-way ANOVA with Dunnett's or Bonfferoni's multiple comparison post test. Statistics were calculated using Prism 5.0. Error bars are representative of the standard deviation.

## Supporting Information

Figure S1 **Serum starvation and colchicine treatment induces growth arrest in MCF-7 cells**. MCF-7 cells (a) untreated, (b) serum-starved, or (c) colchicine (0.1µM)-treated for 24 hours were subjected to cell cycle analysis by flow cytometry. Histograms were generated by plotting log cyclin A-FITC(FL-1) against propidium iodide (FL-3). A = G0/G1, B = S phase, C = G2 and D = M phase. Data is representative of three independent experiments performed in duplicate.
(TIF)

Figure S2 **Densitometry of FOXO3a expression in extract treated MCF-7 and MDA-MD-231 cells presented in** **figure 5c and 5d** **.** (a) MCF-7 cells and (b) MDA-MB-231 cells were transfected with and without 5nM FOXO3 siRNA for 24 hours prior to 2mg/ml extract treatment for up to 24 hours. Cell lysates were collected and FOXO3a protein expression was assessed by western blot. β-actin was used as a loading control. Data is expressed as a fold change in FOXO3a density normalised to β-actin. Data denoted * (p<0.05) and *** (p<0.001) is significant compared to siRNA treated control (time  = 0 hours). Data denoted + (p<0.01) and ++ (p<0.001) is significant compared to untreated control (time  = 0 hours). All data was analysed by one-way ANOVA with Dunnett's multiple comparison post test. Data is representative of three independent experiments.
(TIF)
