# Correction: An Aqueous Extract of *Fagonia cretica* Induces DNA Damage, Cell Cycle Arrest and Apoptosis in Breast Cancer Cells via FOXO3a and p53 Expression

**DOI:** 10.1371/journal.pone.0102655

**Published:** 2014-07-30

**Authors:** Matt Lam, Kirsten Wolff, Helen Griffiths, Amtul Carmichael

In our article entitled “An Aqueous Extract of *Fagonia cretica* Induces DNA Damage, Cell Cycle Arrest and Apoptosis in Breast Cancer Cells via FOXO3a and p53 Expression” (1), we described the effects of plant material from Pakistan that we referred to as *Fagonia cretica*. Linnaeus in Species Plantarum (2) described three species, namely, *cretica*, *arabica* and *hispanica*, while we now have over 160 names published in the genus (3, 4). The circumscription of species in *Fagonia* is reported to be complex due to variability of morphological characters (3, 4).

After the publication of our article it was raised to our attention that in light of the widespread distribution of *F. indica* and the absence from Pakistan of the more narrowly distributed species *F. cretica* the plant material may originate from *F. indica* and not *F. cretica* as indicated in the article. Some reports suggest that in Pakistan that the name *F. cretica* is used as name for what actually is *F. indica* var. *schweinfurthii* Hadidi (5). Recent studies on *Fagonia* elucidated relationships within the genus using morphological and molecular data (3,4). In light of this, we completed a series of DNA sequencing analyses on different batches of plant material used in our studies in order to establish if the material originated from *F. indica* or *F. cretica*. These additional analyses have confirmed that the material used in the study originated from *F*. cf. *indica*.

The methodology for the analyses was as follows: DNA was extracted from batches of plant material using a Qiagen DNeasy Plant minikit, following manufacturer’s instructions. The chloroplast trnLeu intragenic spacer was amplified using primers trnL-c and trnL-d (6), and the the rDNA Internal Transcribed Spacer region was amplified using primers ITS4 and ITS5 (7). Amplification, purification and sequencing were performed as described by Houston and Wolff (8). Sequencing was carried out with the Big Dye Terminator cycle sequencing kit v. 3.1 (Applied Biosystems) according to manufacturer’s instructions. Sequencing products were purified using a Genetix column purification and the sequences of these samples were visualised using an ABI 3100 automated sequencer (Applied Biosystems).

The resulting sequences, both trnL (439 bases) and ITS (489 bases), were blasted to sequences available on GenBank and showed the highest similarity with those of *F. indica* and of *F. paulayana*, while being clearly different from those for *F. cretica* (Table 1). The alignment shows that for trnL the query sample differed from *F. indica* only for the number of repeats in two A-repeats, and one nucleotide substitution with *F. paulayana*. The trnL sequence differed from the *F. cretica* sequences for 12 substitutions and 6 insertions/deletions (Table 2). The ITS query sample differed from *F. indica* and *F. paulayana* for 1 and 3 substitutions, respectively, while the difference with *F. cretica* was 36 substitutions and 1 insertion/deletion (Table 3). The alignment of ITS showed ambiguities at certain positions; this is most likely due to a well-known phenomenon of incomplete sequence conversion between the rDNA paralogs, causing intra-individual sequence diversity. Beier et al. (4) describe that *F. indica* and *F. paulayana* occur sympatrically and cannot be distinguished on the basis of sequence data, but that a single morphological character (persistence of sepals) discriminates the two species.

**Table 1:** Sequence similarity of our query sample and most similar sequences available at Genbank for gene for trn-Leu and rDNA Internal Transcribed Spacer.

*trn Leu*			*Internal transcribed spacer rDNA*		
*Species*	*Similarity*	*Genbank nr*	*Species*	*Similarity*	*Genbank nr*
*F. indica*	99%	AY641593.1	*F. indica*	99%	AY641631.1
*F. indica*	99%	AY300769.1	*F. paulayana*	99%	AY641652.1
*F. indica*	99%	AY641592.1	*F. indica*	99%	AY641630.1
*F. paulayana*	99%	AY641607.1	*F. subinermis*	99%	AY641642.1
*F. paulayana*	98%	AY641606.1	*F. paulayana*	99%	AY641650.1
*F. subinermis*	98%	AY641610.1	*F. indica*	99%	AY641632.1
*F. mahrana*	96%	AY641600.1	*F. paulayana*	99%	AY641654.1
*F. lahovarii*	96%	AY641596.1	*F. mahrana*	98%	AY641639.1
*F. hadramautica*	95%	AY641590.1	*F. gypsophila*	98%	AY641627.1
*F. gypsophila*	95%	AY641589.1	*F. lahovarii*	97%	AY641635.1
*F. acerosa*	95%	AY641579.1	*F. latistipulata*	97%	AY641636.1
*F. indica*	95%	AY300770.1	*F. scabra*	97%	AY641645.1
*F. harpago*	95%	AY641591.1	*F. charoides*	97%	AY641621.1
*F. longispina*	94%	AY641599.1	*F. minutistipula*	96%	AY641641.1
*F. latistipulata*	94%	AY641598.1	*F. longispina*	96%	AY641637.1
*F. bruguieri*	94%	AY641582.1	*F. glutinosa*	96%	AY641626.1
*F. minutistipula*	93%	AY300771.1	*F. bruguieri*	96%	AY641619.1
*F. cretica*	92%	AJ387942.1	*F. olivieri*	96%	AY641646.1
*F. densa*	92%	AY641587.1	*F. mollis*	95%	AY641643.1
*F. laevis*	92%	AY641594.1	*F. bruguieri*	95%	AY641620.1
*F. villosa*	92%	AY641611.1	*F. rangei*	95%	AY641647.1
*F. mollis*	92%	AY641601.1	*F. harpago*	95%	AY641629.1
*F. laevis*	92%	AY641595.1	*F. acerosa*	95%	AY641617.1
*F. scabra*	92%	AY300768.1	*F. luntii*	95%	AY641638.1
*F. charoides*	91%	AY641583.1	*F. laevis*	95%	AY641634.1
*F. orientalis*	91%	AY641603.1	*F. laevis*	95%	AY641633.1
*F. rangei*	91%	AY641609.1	*F. palmeri*	95%	AY641653.1
*F. cretica*	91%	AY641585.1	*F. orientalis*	94%	AY641648.1
*F. arabica*	91%	AY641580.1	*F. boveana*	94%	KF850598.1
*sF. cretica*	91%	AY641586.1	*F. pachyacantha*	94%	AY641651.1
*F. pachyacantha*	90%	AY641604.1	*F. pachyacantha*	94%	AY641649.1
*F. pachyacantha*	90%	AY300772.1	*F. villosa*	94%	AY641640.1
*F. zilloides*	90%	AY641612.1	*F. hadramautica*	94%	AY641628.1
			*F. arabica*	93%	AY641618.1
			*F. chilensis*	93%	AY641622.1
			*F. densa*	93%	AY641625.1
			*F. cretica*	93%	AY641624.1
			*F. scoparia*	90%	AY641644.1
			*F. cretica*	90%	AY641623.1

**Table 2:** Aligned sequences of the trn-Leu gene intron for our query sample and a selection of the most similar sequences and most distant sequences within the genus *Fagonia* available at Genbank.

	10	20	30	40	50	60
**trnL intron query**	GTGATCACTT	TCAAATTCAG	AGAAACCCTG	GAATTAGAAA	TGGGCAATCC	TGAGCCAAAT
**AJ387943 F.indica**	GTGATCACTT	TCAAATTCAG	AGAAACCCTG	GAATTATAAA	TGGGCAATCC	TGAGCCAAAT
**AY641592 F.indica**	–––––––CTT	TCNAATTCAG	AGAANCCCTG	GAATTAGAAA	TGGGCAATCC	TGAGCCAAAT
**AY641608 F.paulayana**	GTGATCACTT	TCAAATTCAG	AGAAACCCTG	GAATTAGAAA	TGGGCAATCC	TGAGCCAAAT
**AY641610 F.subinermi**	GTGATCACTT	TCAAATTCAG	AGAA-CCCTG	GAATTASAAA	TGGGCAATCC	TGAGCCAAAT
**AY641600 F.mahrana**	GTGATCACTT	TCAAATTCAG	AGAAACCCTG	GAATTAGAAA	TGGGCAATCC	TGAGCCAAAT
**AY641596 F.lahovarii**	––GATCACTT	TCAAATTCAG	AGAAACCCTG	GAATTAGAAA	TGGGCAATCC	TGAGCCAAAT
**AJ387942 F.cretica**	GTGATCACTT	TCAAATTCAG	AGAAACCCTG	GAATTAGAAA	TGGGCAATCC	TGAGCCAAAT

	70	80	90	100	110	120
**trnL intron query**	CCTGTTTTCC	TAAAAAAAAA	AAAA––GAAG	TCAATTA–––	–––TAAAATC	AAAAAAGTAA
**AJ387943 F.indica**	CCTGTTTTCC	TAAAAAAAAA	AAAAA–GAAG	TCAATT––––	–––TAAAATC	AAAAAAGTAA
**AY641592 F.indica**	CCTGTTTTCC	TAAAAAAAAA	AAA–––GAAG	TCAATTA–––	–––TAAAATC	AAAAAAGTAA
**AY641608 F.paulayana**	CCTGTTTTCC	TAAAAAAAAA	AAAA––GAAG	TCAATTA–––	–––TAAAATC	AAAAAAGTAA
**AY641610 F.subinermi**	CCTGTTTTCC	TAAAAAAAAA	A–––––GAAG	TCAATTA–––	–––TAAANTC	AAAAAAGTAA
**AY641600 F.mahrana**	CCTGTTTTCC	TAAAAAAAAA	AAAAAAGAAG	TCAATTA–––	–––TAAAATC	AAAAAAGTAA
**AY641596 F.lahovarii**	CCTGTTTTCC	TAAAAAAAAA	AAAA––GAAG	TCAATTAAAA	GAATAAAATC	AAAAAAGTAA
**AJ387942 F.cretica**	CCTGTTTTCC	TAAAAAAAAA	AAA–––GAAG	TAAATCAAAA	GAATCAAATC	AAAAAAGTAA

	130	140	150	160	170	180
**trnL intron query**	AA–––AAAAA	GGATAGGTGC	AGAGACTCAA	TGGAAGCTGT	TCTAAAAAAT	GGAGTTGACT
**AJ387943 F.indica**	AA–––AAAA–	GGATAGGTGC	AGAGACTCAA	TGGAAGCTGT	TCTAAAAAAT	GGAGTTGACT
**AY641592 F.indica**	AA–––AAAA–	GGATAGGTGC	AGAGACTCAA	TGGAAGCTGT	TCTAAAAAAT	GGAGTTGACT
**AY641608 F.paulayana**	AA–––AAAA–	GGATAGGTGC	AGAGACTCAA	TGGAAGCTGT	TCTAAAAAAT	GGAGTTGACT
**AY641610 F.subinermi**	AA–––AAAA–	GGATAGGTGC	AGAGACTCAA	TGGAAGCTGT	TCTAAAAAAT	GGAGTTGACT
**AY641600 F.mahrana**	AA–––AAAA–	GGATGGGTGC	AGAGACTCAG	TGGAAGCTGT	TCTAAAAAGT	GGAGTTGACT
**AY641596 F.lahovarii**	AA–––AAAA–	GGATAGGTGC	AGAGACTCAA	TGGAAGCTGT	TCTAAAAAAT	GGAGTTGACT
**AJ387942 F.cretica**	AATAAAAAAA	GGATAGGTGC	AGAGACTCAA	CGGAAGCTGT	TCTAAAAAAT	GGAGTTGACT

	190	200	210		220	230	240
**trnL intron query**	ACTGCATTAC	GTTAGTCAAG	TCAAGGAATC	CTTGCATCGA		AACTTTTTCA	A–––––––GG
**AJ387943 F.indica**	ACTGCATTAC	GTTAGTCAAG	TCAAGGAATC		CTTGCATCGA	AACTTTTTCA	AA––––––GG
**AY641592 F.indica**	ACTGCATTAC	GTTAGTCAAG	TCAAGGAATC		CTTGCATCGA	AACTTTTTCA	A–––––––GG
**AY641608 F.paulayana**	ACTGCATTAC	GTTAGTCAAG	TCAAGGAATC		CTTGCATCGA	AACTTTTTCA	A–––––––GG
**AY641610 F.subinermi**	ACTGCATTAC	GTTAGTCAAG	TCAAGGAATC		CTTGCATCGA	AACTTTTTCA	A–––––––GG
**AY641600 F.mahrana**	ACTGCATTAC	GTTAGTCAAG	TCAAGGAATC		CTTGCATCGA	AACTTTTTCA	A–––––––GG
**AY641596 F.lahovarii**	ACTGCATTAC	GTTAGTCAAG	TCAAGGAATC		CTTGCATCGA	AACTTTTTCA	A–––––––GG
**AJ387942 F.cretica**	GCTGCATTAC	GTTAGTCAAG	TCAAGGAATC		CTTGCATCGA	AACTTTTTCA	AAGATAAAGG

	250	260	270	280	290	300
**trnL intron query**	ATAACCTTTC	TTTCTATCAA	ACTCTAAAAA	TAAAAAATAT	AAAGACTTTT	TAAATTAATT
**AJ387943 F.indica**	ATAACCTTTC	TTTCTATCAA	ACTCTAAAAA	TAAAAAATAT	AAAGACTTTT	TAAATTAATT
**AY641592 F.indica**	ATAACCTTTC	TTTCTATCAA	ACTCTAAAAA	TAAAAAATAT	AAAGACTTTT	TAAATTAATT
**AY641608 F.paulayana**	ATAACCTTTC	TTTCTATCAA	ACTCTAAAAA	TAAAAAATAT	AAAGACTTTT	TGAATTAATT
**AY641610 F.subinermi**	ATAACCTTTC	TTTCTATCAA	ACTCTAAAAA	TAAAAAATAT	AAAGACTTTT	TCAATTAATT
**AY641600 F.mahrana**	ATAACCTTTC	TTTCTATCAA	ACTATAAAAA	TAAAAAATAT	AAAGACTTTT	T–––––––––
**AY641596 F.lahovarii**	ATAACCTTTC	TTTCTATCAA	ACTCTAAAAA	TAAAAAATAT	AAAGACTTTT	T–––––––––
**AJ387942 F.cretica**	ATAACCTTTT	TTTCTATCAA	ACTCTCAAAA	TAAAAAAGAT	AAAGACTTTT	––––––––––

	310	320	330	340	350	360
**trnL intron query**	GGAATTAATT	GGAAGTTGAA	GAAAGAATCA	AATATGATTT	TATCAAATCA	TTTACTCCAA
**AJ387943 F.indica**	GGAATTAATT	GGAAGTTGAA	GAAAGAATCA	AATATGATTT	TATCAAATCA	TTTACTCCAA
**AY641592 F.indica**	GGAATTAATT	GGAAGTTGAA	GAAAGAATCA	AATATGATTT	TATCAAATCA	TTTACTCCAA
**AY641608 F.paulayana**	GGAATTAATN	GGAAGTTGAA	GAAAGAATCA	AATATGATTT	TATCAAATCA	TTTACTCCAA
**AY641610 F.subinermi**	GGAATTAATT	GGAAGTTGAA	GAAAGAATCA	AATATGATTT	TATCAAATCA	TTTACTCCAA
**AY641600 F.mahrana**	- GAATTAATT	GGAAGTTGAA	GAAAGAATCA	AATATGATTT	TATCAAATCA	TTTACTCCAA
**AY641596 F.lahovarii**	- GAATTAATT	GGAAGTTGAA	GAAAGAATCA	AATATGATTT	TATCAAATCA	TTTACTCCAA
**AJ387942 F.cretica**	GGAATCAATT	GGAAGTTTAA	GAAAGAATCA	AATATGATTT	TATCAAATCA	TTTACTCCAA

	370	380	390	400	410	420
**trnL intron query**	AGTCTTATAT	ATCTTTTTCA	AAAAA-GATT	AGTCAGACGA	GAATAAAGAT	AGAGTCCCAT
**AJ387943 F.indica**	AGTCTTATAT	ATCTTTTTCA	AAAAA-GATT	AGTCAGACGA	GAATAAAGAT	AGAGTCCCAT
**AY641592 F.indica**	AGTCTTATAT	ATCTTTTTCA	AAAAA-GATT	AGTCAGACGA	GAATAAAGAT	AGAGTCCCAT
**AY641608 F.paulayana**	AGTCTTATAT	ATCTTTTTCA	AAAAA-GATT	AGTCAGACGA	GAATAAAGAT	AGAGTCCCAT
**AY641610 F.subinermi**	AGTCTTATAT	ATCTTTTTCA	AAAAA-GATT	AGTCAGACGA	GAATAAAGAT	AGAGTCCCAT
**AY641600 F.mahrana**	AGTCTTATAT	ATCTTTTTCA	AAAAA-GATT	AGTCAGACGA	GAATAAAGAT	AGAGTCCCAT
**AY641596 F.lahovarii**	AGTCTTATAT	ATCTTTTTCA	AAAAA-GATT	AGTCAGACGA	GAATAAAGAT	AGAGTCCCAT
**AJ387942 F.cretica**	AGTCTGATAT	ATCTTTTTCA	AAAAAAGATT	AGTCAGGCGA	GAATAAAGAT	AGAGTCCCAT

	430	440	450	460	470	480
**trnL intron query**	TCTATATGTC	AATATTGACA	ACAATGAAAT	TTATAGTGAG	AGGAAAATCC	GTCGATTTTA
**AJ387943 F.indica**	TCTATATGTC	AATATTGACA	ACAATGAAAT	TTATAGTGAG	AGGAAAATCC	GTCGATTTTA
**AY641592 F.indica**	TCTATATGTC	AATATTGAC-	––––––––––	––––––––––	––––––––––	––––––––––
**AY641608 F.paulayana**	TCTATATGTC	AATATTGAC-	––––––––––	––––––––––	––––––––––	––––––––––
**AY641610 F.subinermi**	TCTATATGTC	AATATTGAC-	––––––––––	––––––––––	––––––––––	––––––––––
**AY641600 F.mahrana**	TCTATATGTC	AATATTGAC-	––––––––––	––––––––––	––––––––––	––––––––––
**AY641596 F.lahovarii**	TCTATATGTC	AATATTGAC-	––––––––––	––––––––––	––––––––––	––––––––––
**AJ387942 F.cretica**	TCTATATGTC	AATATTGACA	ACAATGAAAT	TTATAGTGAG	AGGAAAATCC	GTCGATTTTA

	490	
**trnL intronquery**	TCAATCGTGA	GGGT
**AJ387943 F.indica**	TCAATCGTGA	GGG–
**AY641592 F.indica**	––––––––––	––––
**AY641608 F.paulayana**	––––––––––	––––
**AY641610 F.subinermi**	––––––––––	––––
**AY641600 F.mahrana**	––––––––––	––––
**AY641596 F.lahovarii**	––––––––––	––––
**AJ387942 F.cretica**	TCAATGTGGA	GGGT

**Table 3:** Aligned sequences of the rDNA Internal Transcribed Spacer for our query sample and a selection of the most similar sequences and most distant sequences within the genus *Fagonia* available at Genbank.

		10	20	30	40	50	60
**query**	**ITS**	AGAGCATACC	CCTTCCTCGA	GTGTCGGGAG	GGAGACTTCC	TGACATTATA	ACGAACCCCG
**AY641631**	**F.indica**	AGAGCATACC	CCTTCCTCGA	GTGTCGGGAG	GGAGACTTCC	TGACATTATA	ACGAACCCCG
**AY641630**	**F.indica**	AGAGCATACC	CCTTCCTNGA	GTGTCGGGAG	GGAGACTTCC	NGACATTATA	ACGAACCCCG
**AY641632**	**F.indica**	AGAGCATACC	CCTTCCTCGA	GTGTCGGGAG	GGAGACTTCN	TGACATTATA	ACGAACCCCG
**AY641652**	**F.paulayana**	AGAGCATACC	CCTTCCTCGA	GTGTCGGGAG	GGAGACTTCC	TGACATTATA	ACGAACCCCG
**AY641650**	**F.paulayana**	AGAGCATACC	CCTTCCTCGA	GTGTCGGGAG	GGAGACTTCC	TGACATTATA	ACGAACCCCG
**AY641654**	**F.paulayana**	AGAGCATACC	CCTTCCTCGA	GTGTCNGGAG	GGAGACTTCN	TGACATTATA	ACGAACCCCG
**AY641642**	**F.subinermi**	AGAGCATACC	CCTTCCTCGA	GTGTCAGGAG	GGAAACTTCC	TGACATTATA	ACGAACCCCG
**AY641639**	**F.mahrana**	AGAGCATGCC	CCTTCCTCGA	GTGTCGGGAG	GGAGACTTCN	TGACATCATA	ACGAACCCCG
**AY641627**	**F.gypsophil**	AGAGCATACC	CCTTCCTCGA	GTGTCAGGAG	GGAGACTTCC	TGACATTATA	ACGAACCCCG
**AY641635**	**F.lahovarii**	--AGCATGCC	CCTTCCTCGA	GTGTTGGGAG	GGAGACTTCC	CGACATCATA	ACGAACCCCG
**AY641636**	**F.latistipu**	AGAGCATGCC	CCTTCCTCGA	GTGTTGGGAG	GGAGACTTCC	CGACATCATA	ACGAACCCCG
**AY641641**	**F.minutisti**	AGAGCATGCC	CCTTCCTCGA	GTGTCGGGAG	GGAGACTTCC	CGACATCATA	ACGAACCCCG
**AY641624**	**F.cretica**	AGAGCATGCC	CCTTCATCGA	GTGTTGAGAG	GGAGACCTCT	CGACATCATA	ACGAACCCCG
**AY641623**	**F.cretica**	AGAGCATGCC	CCTTCATCGA	GTGTTGAGAG	GGAGACCTCT	CGACATCATA	ACGAACCCCG

		70	80	90	100	110	120
**query**	**ITS**	GCGTGAAAAA	CGCCAAGGAA	AACAAACAAA	AAA-GGAGAC	TGCGTTCGCG	TGGCCTCCTT
**AY641631**	**F.indica**	GCGTGAAAAA	CGCCAAGGAA	AACAAACAAA	AAA-GGAGAC	TGCGTTCGCG	TGGCCTCCTT
**AY641630**	**F.indica**	GCNTGAAAAA	CGCCANGGAA	ANCAAACAAA	AAA-GGAGAC	TGCGTTCGCG	TGGCCTCCTT
**AY641632**	**F.indica**	GCGTGAAAAM	CGCCAAGGAA	AMCAAMCAAA	AAA-GGARAC	TGCGTTCGCG	TGGCCTCCTT
**AY641652**	**F.paulayana**	GCGTGAAAAA	CGCCAAGGAA	AACAAACAAA	AANAGGAGAC	TGCGTTCGCG	TGGCCTCCTT
**AY641650**	**F.paulayana**	GCGTGAAAAA	CGCCAAGGAA	AACAAACAAA	TAA-GGAGAC	TGCGTTCGCG	TGGCCTCCTT
**AY641654**	**F.paulayana**	GCGTGAAAAA	CGCCAAGGAA	AACAAACAAA	TAN-GGAGAC	TGCGTTCGCG	TGGCCTCCTT
**AY641642**	**F.subinermi**	GCGTGAAAAA	CGCCAAGGAA	AACAAACAAA	AAA-GGAGAC	TGCGTTCGCG	TGGCCTCCTT
**AY641639**	**F.mahrana**	GCGTGAAAAA	CGCCAAGGAA	AACAAACAAA	CAA-GGAGAT	TGCGTTCGCG	TRGCCTCCTT
**AY641627**	**F.gypsophil**	GCGTGAAAAA	CGCCAAGGAA	AACAAACAAA	CAA-GGAGAC	TGCGTTCGCG	TGGCCTCCTT
**AY641635**	**F.lahovarii**	GCGTGAAAAA	CGCCAAGGAA	AACAAACAAA	CAA-GGAGAT	TGCGTCCGCG	TGGCCTCCTT
**AY641636**	**F.latistipu**	GCGTGAAAAA	CGCCAAGGAA	AACAAACAAA	TAA-GGAGAT	TGCGTCCGCG	TGACCTCCTT
**AY641641**	**F.minutisti**	GCGTTAAAAG	CGCCAAGGAA	AACAAACAAA	ACAAGGAGAC	TGCGTTCGCG	TGGCCTCCTT
**AY641624**	**F.cretica**	GCGTGAAAAG	CGCCAAGGAA	AACAAACAAA	ACAAGGAGAT	TGCATTCGTG	TGGCCTCCTT
**AY641623**	**F.cretica**	GCGTGAAAAG	CGCCAAGGAA	AACAAACAAA	ACAAGGAGAT	TACATTCGTG	TGGCCTCCTT

		130	140	150	160	170	180
**query**	**ITS**	TGTATTGAAT	CAAAATGACT	CTCGGCAACG	GATATCTCGG	CTCTCGCATC	GATGAAGAAC
**AY641631**	**F.indica**	TGTATTGAAT	CAAAATGACT	CTCGGCAACG	GATATCTCGG	CTCTCGCATC	GATGAAGAAC
**AY641630**	**F.indica**	TGTATTGAAT	CAAAATGACT	CTCGGCAACG	GATATCTCGG	CTCTCGCATC	GATGAAGAAC
**AY641632**	**F.indica**	TGTATTGAAT	CAAAATGACT	CTCGGCAACG	GATATCTCGG	CTCTCGCATC	GATGAAGAAC
**AY641652**	**F.paulayana**	TGTATTGAAT	CAAAATGACT	CTCGGCAACG	GATATCTCGG	CTCTCGCATC	GATGAAGAAC
**AY641650**	**F.paulayana**	TGTATTGAAT	CAAAATGACT	CTCGGCAACG	GATATCTCGG	CTCTCGCATC	GATGAAGAAC
**AY641654**	**F.paulayana**	TGTATTGAAT	CAAAATGACT	CTCGGCAACG	GATATCTCGG	CTCTCGCATC	GATGAAGAAC
**AY641642**	**F.subinermi**	TGTATTGAAT	CAAAATGACT	CTCGGCAACG	GATATCTCGG	CTCTCGCATC	GATGAAGAAC
**AY641639**	**F.mahrana**	TGTATTGAAT	CAAAATGACT	CTCGGCAACG	GATATCTCGG	CTCTCGCATC	GATGAAGAAC
**AY641627**	**F.gypsophil**	TGTATTGAAT	CAAAATGACT	CTCGGCAACG	GATATCTCGG	CTCTCGCATC	GATGAAGAAC
**AY641635**	**F.lahovarii**	TGCATTGAAT	CAAAATGACT	CTCGGCAACG	GATATCTCGG	CTCTCGCATC	GATGAAGAAC
**AY641636**	**F.latistipu**	TGCATTGAAT	CAAAATGACT	CTCGGCAACG	GATATCTCGG	CTCTCGCATC	GATGAAGAAC
**AY641641**	**F.minutisti**	TGCATTGAGT	CAAAACGACT	CTCGGCAACG	GATATCTCGG	CTCTCGCATC	GATGAAGAAC
**AY641624**	**F.cretica**	TGCATTGAAT	CAAAACGACT	CTCGGCAACG	GATATCTCGG	CTCTCGCATC	GATGAAGAAC
**AY641623**	**F.cretica**	TGCATTGAAT	CAAAACGACT	CTCGGCAACG	GATATCTCGG	CTCTCGCATC	GATG––––––

		190	200	210	220	230	240
**query**	**ITS**	GTAGCGAAAT	GCGATACTTG	GTGTGAATTG	CAGAATCCAG	TGAACCATCG	AGTCTTTGAA
**AY641631**	**F.indica**	GTAGCGAAAT	GCGATACTTG	GTGTGAATTG	CAGAATCCAG	TGAACCATCG	AGTCTTTGAA
**AY641630**	**F.indica**	GTAGCGAAAT	GCGATACTTG	GTGTGAATTG	CAGAATCCAG	TGAACCATCG	AGTCTTTGAA
**AY641632**	**F.indica**	GTAGCGAAAT	GCGATACTTG	GTGTGAATTG	CAGAATCCAG	TGAACCATCG	AGTCTTTGAA
**AY641652**	**F.paulayana**	GTAGCGAAAT	GCGATACTTG	GTGTGAATTG	CAGAATCCAG	TGAACCATCG	AGTCTTTGAA
**AY641650**	**F.paulayana**	GTAGCGAAAT	GCGATACTTG	GTGTGAATTG	CAGAATCCAN	NGAACCATCG	AGTCTTTGAA
**AY641654**	**F.paulayana**	GTAGCGAAAT	GCGATACTTG	GTGTGAATTG	CAGAATCCAG	TGAACCATCG	AGTCTTTGAA
**AY641642**	**F.subinermi**	GTAGCGAAAT	GCGATACTTG	GTGTGAATTG	CAGAATCCAG	TGAACCATCG	AGTCTTTGAA
**AY641639**	**F.mahrana**	GTAGCGAAAT	GCGATACTTG	GTGTGAATTG	CAGAATCCAG	YGAACCATCG	AGTCTTTGAA
**AY641627**	**F.gypsophil**	GTAGCGAAAT	GCGATACTTG	GTGTGAATTG	CAGAATCCAG	CGAACCATCG	AGTCTTTGAA
**AY641635**	**F.lahovarii**	GTAGCGAAAT	GCGATACTTG	GTGTGAATTG	CAGAATCCAG	TGAACCATCG	AGTCTTTGAA
**AY641636**	**F.latistipu**	GTAGCGAAAT	GCGATACTTG	GTGTGAATTG	CAGAATCCAG	TGAACCATCG	AGTCTTTGAA
**AY641641**	**F.minutisti**	GTAGCGAAAT	GCGATACTTG	GTGTGAATTG	CAGAATCCAG	CGAACCATCG	AGTCTTTGAA
**AY641624**	**F.cretica**	GTAGCGAAAT	GCGATACTTG	GTGTGAATTG	CAGAATCCAG	CGAACCATCG	AGTCTTTGAA
**AY641623**	**F.cretica**	–––––––––T	GCGATACTTG	GTGTGAATTG	CAGAATCCAG	CGAACCATCG	AGTCTTTGAA

		250	260	270	280	290	300
**query**	**ITS**	CGCAAGTTGC	GCCCGAAGCC	TTCGGGCTAA	GGGCACGTCT	GCCTGGGTGT	CACGCAATGT
**AY641631**	**F.indica**	CGCAAGTTGC	GCCCGAAGCC	TTCGGGCTAA	GGGCACGTCT	GCCTGGGTGT	CACGCAATGT
**AY641630**	**F.indica**	CGCAAGTTGC	GCCCGAAGCC	TTCGGGCTAA	GGGCACGTCT	GCCTGGGTGT	CACGCAATGT
**AY641632**	**F.indica**	CGCAAGTTGC	GCCCGAAGCC	TTCGGGCTAA	GGGCACGTCT	GCTTGGGTGT	CACGCAATGT
**AY641652**	**F.paulayana**	CGCAAGTTGC	GCCCGAAGCC	TTCGGGCTAA	GGGCACGTCT	GCCTGGGTGT	CACGCAATGT
**AY641650**	**F.paulayana**	CGCAAGTTGC	GCCCGAAGCN	TTCGGGCTAA	GGGCACGTCT	GCNTGGGTGT	CACGCAATGT
**AY641654**	**F.paulayana**	CGCAAGTTGC	GCCCGAAGCC	TTCGGGCTAA	GGGCACGTCT	GCCTGGGTGT	CACGCAATGT
**AY641642**	**F.subinermi**	CGCAAGTTGC	GCCCGAAGCC	TTCGGGCTAA	GGGCACGTCT	GCCTGGGTGT	CACGCAATGT
**AY641639**	**F.mahrana**	CGCAAGTTGC	GCCCGAAGCC	TTCGGGCTAA	GGGCACGTCT	GCCTGGGTGT	CACGCAATGT
**AY641627**	**F.gypsophil**	CGCAAGTTGC	GCCCGAAGCC	TTCGGGCTAA	GGGCACGTCT	GCCTGGGTGT	CACGCAATGT
**AY641635**	**F.lahovarii**	CGCAAGTTGC	GCCCGAAGCC	TTCGGGCTAA	GGGCACGTCT	GCCTGGGTGT	CACGCAATGT
**AY641636**	**F.latistipu**	CGCAAGTTGC	GCCCGAAGCC	TTCGGGCTAA	GGGCACGTCT	GCCTGGGTGT	CACGCAATGT
**AY641641**	**F.minutisti**	CGCAAGTTGC	GCCCGAAGCC	TTCGGGCTAA	GGGCACGTCT	GCCTGGGTGT	CACGCAACGT
**AY641624**	**F.cretica**	CGCAAGTTGC	GCCCGAAGCC	TTCGGGTTAA	GGGCACGTCT	GCCTGGGTGT	CACGCAATGT
**AY641623**	**F.cretica**	CGCAAGTTGC	GCCCGAAGCC	TTCGGGTTAA	GGGCACGTCT	GCCTGGGTGT	CACGCAATGT

		310	320	330	340	350	360
**query**	**ITS**	CGCTCTCCAC	TCCATATTGA	TTGGATGAGC	GTATGATGGT	CTCCCGTGAG	CTTTCTTTGC
**AY641631**	**F.indica**	CGCTCTCCAC	TNCATATTGA	TTGGATGAGC	GTATGATGGT	CTCCCGTGAG	CTTTCTTTGC
**AY641630**	**F.indica**	CGCTCTCCAC	TCCATATTGA	TTGGATGAGC	GTATGATGGT	CTCCCGTGAG	CTTTCTTTGC
**AY641632**	**F.indica**	CGCTCTCCAC	TCCATATTGA	TTGGATGAGC	GTATGATGGT	CTCCCGTGAG	CTTTCTTTGC
**AY641652**	**F.paulayana**	CGCTCTCCAC	TCCATATTGA	TTGGATGAGC	GTATGATGGT	CTCCCGTGAG	CTTTCTTTGC
**AY641650**	**F.paulayana**	CGCTCTCCAC	TCCATATTGA	TTGGATGAGC	GTATGATGGT	CTCCCGTGAG	CTTTCTTTGC
**AY641654**	**F.paulayana**	CGCTCTCCAC	TCNATATTGA	TTGGATGAGC	GTATGATGGT	CTCCCGTGAG	CTTTCTTTGC
**AY641642**	**F.subinermi**	CGCTCTCCAC	TCCATATTGA	TTGGATGAGC	GTATGATGGT	CTCCCGTGAG	CTTTCTTTGC
**AY641639**	**F.mahrana**	CGCTCTCCAC	TCCATATTGA	TTGGATGAGC	GTATGATGGT	CTCCCGTGAG	CTATCTTTGC
**AY641627**	**F.gypsophil**	CGCTCCCCAC	TCCATATTGA	TTGGATGAGC	GTATGATGGT	CTCCCGTGAG	CTATCTTTGC
**AY641635**	**F.lahovarii**	CGCTCCCCAC	TCTATATTGA	TTGGATGAGC	GTATGATGGT	CTCCCGTGAG	CTATCTNTGC
**AY641636**	**F.latistipu**	CGCTCCCCAC	TCTATATTGA	TTGGATGAGC	GTATGATGGT	CTCCCGCGAG	CAATCTTTGC
**AY641641**	**F.minutisti**	CGCTCCCCAT	TTCATATTGA	TTGGATGAGC	GTATGATGGT	CTCCCGTGAG	CTATATTTGC
**AY641624**	**F.cretica**	CGCTCCCCAC	TCCAAATTGA	TTGGGTGAGT	GTATGATGGT	CTCCCGTGAG	CTATATTTGC
**AY641623**	**F.cretica**	CGCTCCCCAC	TCCAAATTGA	TTGGGTGAGT	GTATGATGGT	CTCCCGTGAG	CTATATTTGC

		370	380	390	400	410	420
**query**	**ITS**	TTGTGGTTGG	CCTAAACATG	AGTCCTCGGY	GCGAATTGTG	CCACGGTCTT	AGGTGGTCGA
**AY641631**	**F.indica**	TTGTGGTTGG	CCTAAACATG	AGTCCTCGGN	GCGAATTGTG	CCACGGTCTT	AGGTGGTCGA
**AY641630**	**F.indica**	TTGTGGTTGG	CCTAAACATG	AGTCCTCGGT	GCGAATTGTG	CCACGGTCTT	AGGTGGTCGA
**AY641632**	**F.indica**	TTGTGGTTGG	CCTAAACATG	AGTCCTCGGT	GCGAATTGTG	CCACGGTCTT	AGGTGGTCGA
**AY641652**	**F.paulayana**	TTGTGGTTGG	CCTAAACATG	AGTCCTCGGT	GTGAATTGTG	CCACGGTCTT	AGGTGGTCGA
**AY641650**	**F.paulayana**	TTGTGGTTGG	CCTAAACATG	AGTCCTCGGN	GCGAATTGTG	CCACGGTCTT	AGGTGGTNGA
**AY641654**	**F.paulayana**	TTGTGGTTGG	CCTAAACATG	AGTCCTCGGN	GCGAATTGTG	CCACGGTCTT	AGGTGGTCGA
**AY641642**	**F.subinermi**	TTGTGGTTGG	CCTAAACATG	AGTCCTCGGT	GCAAATTGTG	CCACGGTCTT	AGGTGGTCGA
**AY641639**	**F.mahrana**	TTGTGGTTGG	CCTAAACATG	AGTCCTCGGC	GCGAATTGTG	CCACGGTCTT	AGGTGGTCGA
**AY641627**	**F.gypsophil**	TTGTGGTTGG	CCTAAACATG	AGTCCTCGGC	GCGAATTGTG	CCACGGTCTT	AGGTGGTCGA
**AY641635**	**F.lahovarii**	TTGTGGTTGG	CCTAAACATG	AGTCCTCGGT	GCGAATTGTG	CCACGGTCTT	AGGTGGTCGA
**AY641636**	**F.latistipu**	TTGTGGTTGG	CCTAAACATG	AGTCCTCGGT	GCGAATTGTG	CCACGGTCTT	AGGTGGTCGA
**AY641641**	**F.minutisti**	TTGTGGTTGG	CCTAAACACG	AGTCCTCGGC	GCGAATTGTG	CCACGGTCTT	AGGTGGTCGA
**AY641624**	**F.cretica**	TTGTGGTTGG	CCTAAACACG	AGTCCTCGGC	ACGGATTGTG	TCACGGTCTT	AGGTGGTCGA
**AY641623**	**F.cretica**	TTGTGGTTGG	CCTAAACACG	AGTCCTCGGC	ACGGATTGTG	TCACGGTCTT	AGGTGGTCGA

		430	440	450	460	470	480
**query**	**ITS**	AACCCTGAAG	GATCGTGTGC	CCATTTTA-G	CCATAGAGAG	GAACTTGCAA	CCCTAGAGCA
**AY641631**	**F.indica**	AACCCTGAAG	GATCGTGTGC	CCATTTTA-G	CCATAGAGAG	GAACTTGCAA	CCCTAGAGCA
**AY641630**	**F.indica**	AACCCTGAAG	GATCGTGTGC	CCATTTTA-G	CCATAGAGAG	GAACTTGCAA	CCCTAGAGCA
**AY641632**	**F.indica**	AACCCTGAAG	GATCGTGTGC	CCATTTTA-G	CCATAGAGAG	GAACTTGCAA	CCCTAGAGCA
**AY641652**	**F.paulayana**	AACCCTGAAG	GATCGTGTGC	CCATTTTA-G	CCATAGAGAG	GAACTTGCAA	CCCTAGAGCA
**AY641650**	**F.paulayana**	AACCCTGAAG	GATCGTGTGC	CCATTTTA-G	CCATAGAGAG	GAACTTGCAA	CCCTAGAGCA
**AY641654**	**F.paulayana**	AACCCTGAAG	GATCGTGTGC	CCATTTTA-G	CCATAGAAAG	GAACTTGCAA	CCCTAGAGCA
**AY641642**	**F.subinermi**	AACCCTGAAG	GATCGTGTGC	CCATTTTA-G	CCATAGAGAG	AAACTTGCAA	CCCTAGAGCA
**AY641639**	**F.mahrana**	AACCCTGAAG	GATCGTGTGC	CCATTTTA-G	CCATAGAGAG	GAACTTGCAA	CCCTAGAGCA
**AY641627**	**F.gypsophil**	AAGCCTGAAG	GATCGTGTGC	CCATTTCA-G	CCATAGAGAG	GAACTTGCAA	CCCTAGAGCA
**AY641635**	**F.lahovarii**	AACCCTGAAG	GATCGTGTGC	CCATTTTA-G	TCATATAGAG	GAACTTGCAA	CCCTAGAGCA
**AY641636**	**F.latistipu**	AACCCTGAAG	GATCGTGTGC	CCATTTTA-G	TCATAGAGAG	GAACTTGTAA	CCCTAGAGCA
**AY641641**	**F.minutisti**	AACCCTGAAG	GATCGTGTGT	CCATTTTA-G	CCATAGAGAG	GAATTTGCAA	CCCTAGAGCA
**AY641624**	**F.cretica**	AACCCTGAAA	GATCGTGTGC	TCATTTCTTG	CCATAGTTAG	GAACTTGCAA	CCCTAAAGCA
**AY641623**	**F.cretica**	AACCCTGAAA	GATCGTGTGC	TCATTTCT-G	CCATAGCTAG	GAACTTGCAA	CCCTAAAGCA

**query**	**ITS**	TGTCGATCT
**AY641631**	**F.indica**	TGTCGATCT
**AY641630**	**F.indica**	TGTCGATCT
**AY641632**	**F.indica**	TGTCGATCT
**AY641652**	**F.paulayana**	TGTNGATCT
**AY641650**	**F.paulayana**	TGTCGATCT
**AY641654**	**F.paulayana**	TGTCGATCT
**AY641642**	**F.subinermi**	TGTCGATCT
**AY641639**	**F.mahrana**	TGTCGATCT
**AY641627**	**F.gypsophil**	TGTCAATCT
**AY641635**	**F.lahovarii**	TGTCGATCT
**AY641636**	**F.latistipu**	TGTCGATCT
**AY641641**	**F.minutisti**	TGTCGAT––
**AY641624**	**F.cretica**	TGTCGATCT
**AY641623**	**F.cretica**	TGTCGATCT

We conclude that *F. indica* or its sister species *F. paulayana* is the genetic identity of the plant material being commonly referred to as *F. cretica* in Pakistan and which has cytotoxic activity towards breast cancer cells. We conclude that it is more likely to be *F. indica* than *F. paulayana* for three reasons. Firstly, our ITS sequence was more similar to that of *F. indica*. Secondly, the trnL sequence only had repeat number differences with *F. indica*, which is a more likely evolutionary step than the substitution that was found in the comparison with *F. paulayana*. Thirdly, the distribution of *F. indica* encompasses the region of origin of our samples. The article title, abstract and main text should therefore refer to *F*. cf. *indica* and not *F. cretica*. The active compound has now been isolated and is undergoing molecular characterization.

We are grateful to Dr. Schori, an expert plant systematist, who alerted us to the likely misidentification of the species.

Kirsten Wolff was responsible for the additional DNA analyses described in this Correction and she has been added as a co-author. The author list should therefore be revised to read as follows:

Matt Lam, Kirsten Wolff, Helen Griffiths, Amtul Carmichael.

Dr. Wolff is affiliated at School of Biology, Newcastle University and has no competing interests in relation to this work.
