# Supplementary figures and images for: An Aqueous Extract of Fagonia cretica Induces DNA Damage, Cell Cycle Arrest and Apoptosis in Breast Cancer Cells via FOXO3a and p53 Expression
Source: PLoS One. 2012 Jun 27;7(6):e40152. doi: 10.1371/journal.pone.0040152 (PMC3384610; doi:10.1371/journal.pone.0040152)

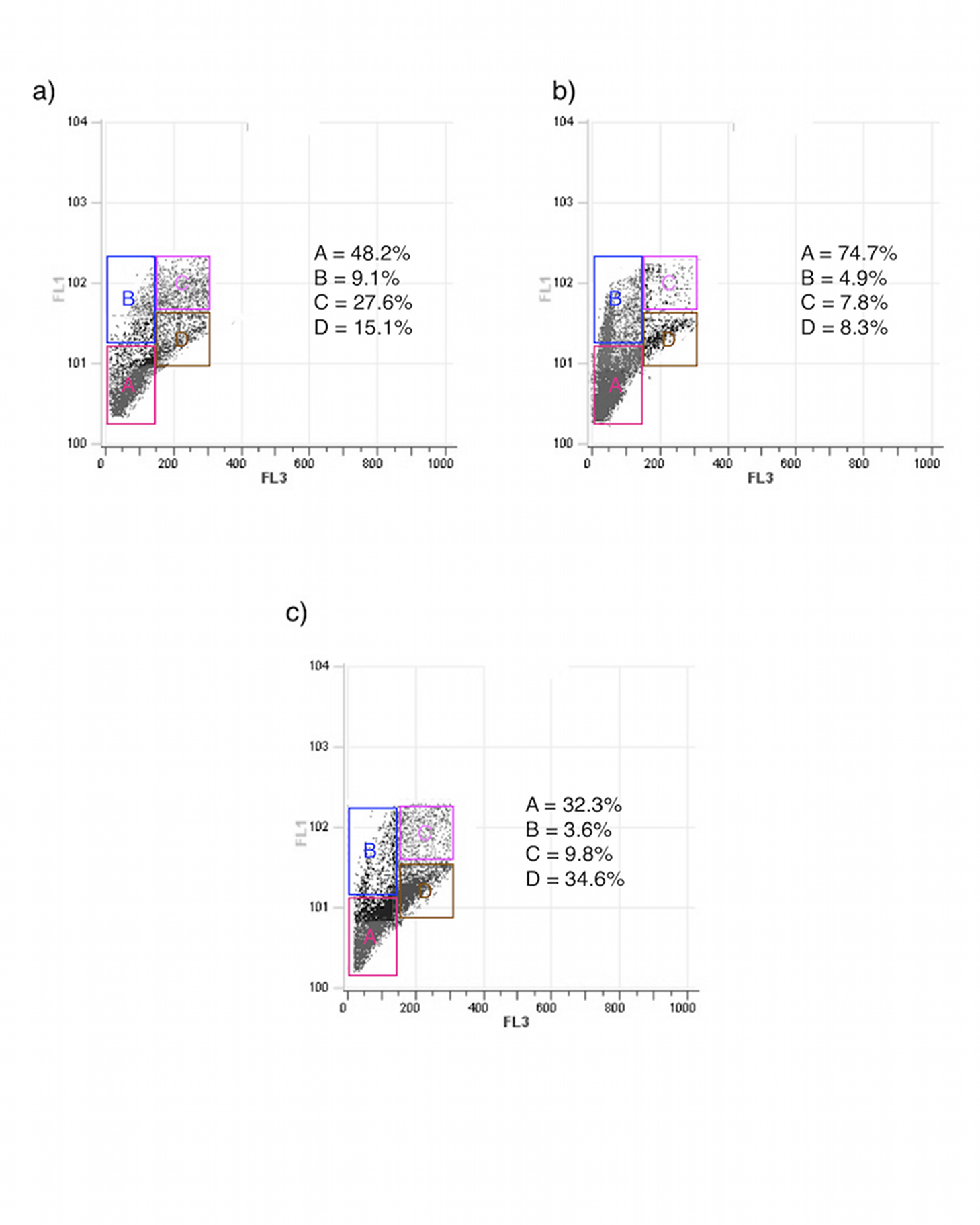

Supplement: Figure S1 — Serum starvation and colchicine treatment induces growth arrest in MCF-7 cells. MCF-7 cells (a) untreated, (b) serum-starved, or (c) colchicine (0.1µM)-treated for 24 hours were subjected to cell cycle analysis by flow cytometry. Histograms were generated by plotting log cyclin A-FITC(FL-1) against propidium iodide (FL-3). A = G0/G1, B = S phase, C = G2 and D = M phase. Data is representative of three independent experiments performed in duplicate. (TIF) [file pone.0040152.s001.tif]

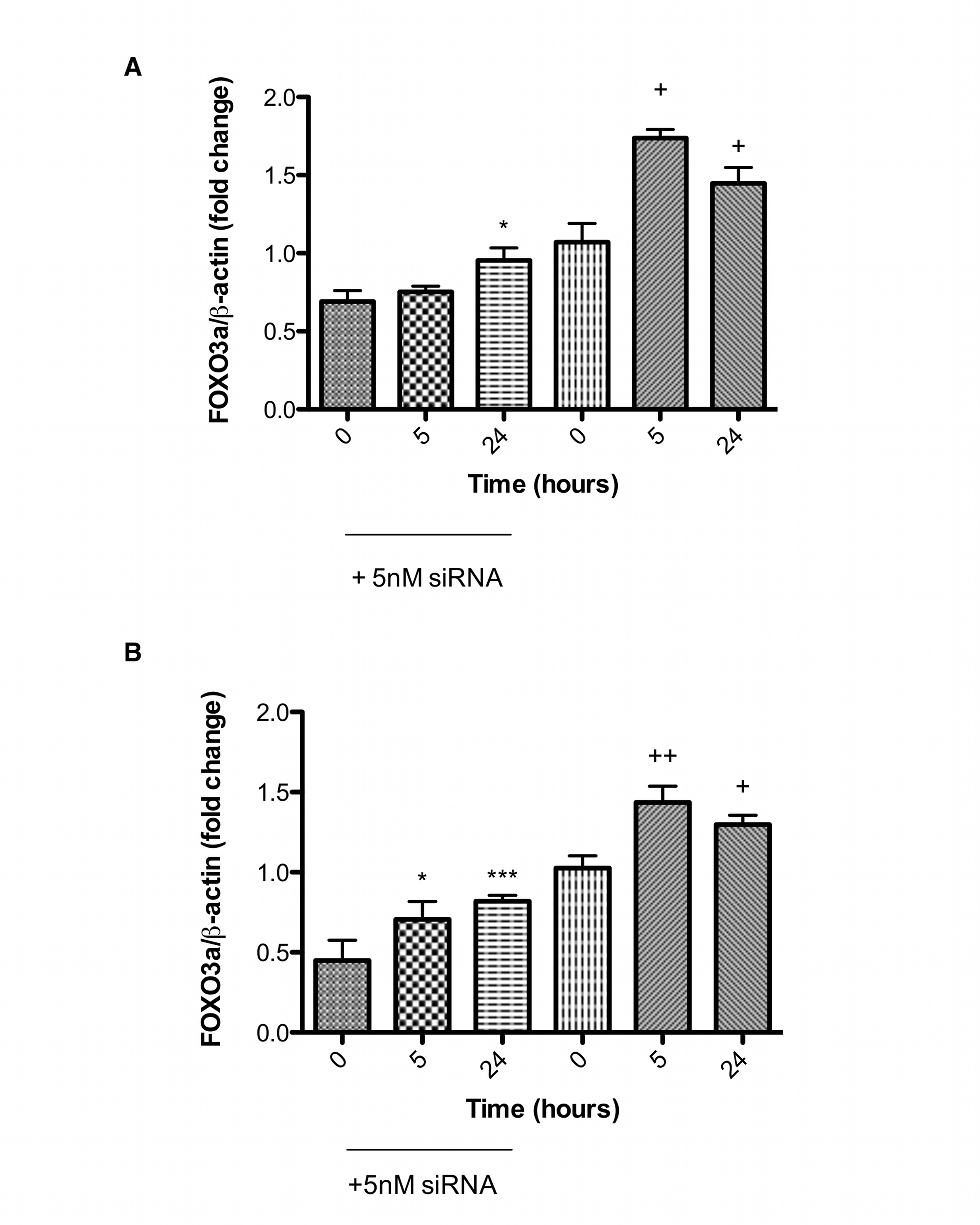

Supplement: Figure S2 — Densitometry of FOXO3a expression in extract treated MCF-7 and MDA-MD-231 cells presented in figure 5c and 5d . (a) MCF-7 cells and (b) MDA-MB-231 cells were transfected with and without 5nM FOXO3 siRNA for 24 hours prior to 2mg/ml extract treatment for up to 24 hours. Cell lysates were collected and FOXO3a protein expression was assessed by western blot. β-actin was used as a loading control. Data is expressed as a fold change in FOXO3a density normalised to β-actin. Data denoted * (p<0.05) and *** (p<0.001) is significant compared to siRNA treated control (time = 0 hours). Data denoted + (p<0.01) and ++ (p<0.001) is significant compared to untreated control (time = 0 hours). All data was analysed by one-way ANOVA with Dunnett's multiple comparison post test. Data is representative of three independent experiments. (TIF) [file pone.0040152.s002.tif]
